# What is binocular disparity?

**DOI:** 10.3389/fpsyg.2014.00870

**Published:** 2014-08-12

**Authors:** Joseph S. Lappin

**Affiliations:** Vanderbilt UniversityNashville, TN, USA

**Keywords:** stereopsis, binocular disparity, depth, binocular vision, surface shape, intensity, image structure

## Abstract

What are the geometric primitives of binocular disparity? The Venetian blind effect and other converging lines of evidence indicate that stereoscopic depth perception derives from disparities of higher-order structure in images of surfaces. Image structure entails spatial variations of intensity, texture, and motion, jointly structured by observed surfaces. The spatial structure of binocular disparity corresponds to the spatial structure of surfaces. Independent spatial coordinates are not necessary for stereoscopic vision. Stereopsis is highly sensitive to structural disparities associated with local surface shape. Disparate positions on retinal anatomy are neither necessary nor sufficient for stereopsis.

## INTRODUCTION: SPATIAL INFORMATION

Stereoscopic vision provides important information about the spatial structure of the surrounding world. The two eyes offer largely similar optical images but from slightly different vantage points. The resulting small disparities between the two monocular images constitute visually important information not available in either image alone. The binocular visual system is extraordinarily sensitive to this stereoscopic information. But what, exactly, is binocular disparity? The issue is not terminology, but the input information. Identifying the input is necessary for determining how that input is processed.

One aspect of this problem is the *“correspondence problem”* — to identify corresponding spatial elements in the two monocular images (Julesz, 1960, 1971; Marr and Poggio, 1976, 1979). The nature and importance of this problem were highlighted by Julesz’s elegant experiments with random-dot stereograms. These random texture patterns contain large numbers of identical elements with countless potential binocular correspondences and disparities. Evidently, the corresponding image features cannot be individual texture elements. Cooperative visual interactions among local texture elements on smooth surfaces seem necessary for stereopsis, as Julesz (1960, 1971) and Marr and Poggio (1976, 1979) emphasized. Research continues on the visual processes that yield correspondence (Blake and Wilson, 2011).

Beyond the correspondence problem, however, binocular disparity involves a representation of spatial structure. Spatial positions of corresponding image features are often represented in relation to hypothetical anatomically defined retinal coordinates; and disparity is represented as a binocular difference in these coordinates. By definition, these retinal coordinates are independent of optical image structure.

This spatial representation is testable, however, with plausible alternative hypotheses. The present article reviews evidence about the spatial structure of binocular disparity. Articles by Lappin and Craft (1997, 2000) and Lappin et al. (2011) are also relevant.

As discussed by Lappin et al. (2011), two psychophysical criteria for identifying information for vision are *resolution* and *invariance*. *Resolution* involves precision of discrimination, limited by variability. In short, what do the two eyes see best? Information and geometric structure are also defined by *invariance* — by the groups of transformations of observational conditions (e.g., viewing position and illumination) under which they remain invariant. Such invariance is experimentally testable.

## IMAGE INTENSITIES AND VISUAL SPACE

### THE VENETIAN BLIND EFFECT

Several phenomena motivate reexamination of binocular disparity. One motivation is the “Venetian blind effect” (VBE, for short) — where dichoptic intensity differences of vertical gratings with non-disparate edges produce a perceived change in 3D surface slant. Apparently, spatial disparity is not necessary.

Cibis and Haber (1951), Ogle (1962), and Howard and Rogers (2002) suggest that the VBE requires no revision of theories of stereopsis: Monocular intensity patterns may affect spatial position signals — because light scattering or nonlinear visual signaling may affect spatial disparity.

Extensive studies by Filley et al. (2011), Hetley and Stine (2011), and Dobias and Stine (2012), however, clearly demonstrate that the VBE derives from disparate intensities not spatial positions. Disparate intensities and edge positions have additive effects on perceived depth; and the two disparities can cancel each other.

The VBE is also consistent with other experimental evidence that disparities in surface highlights and shading contribute to perception of 3D structure (Bülthoff and Mallot, 1988; Norman et al., 1995; Todd et al., 1997; Vuong et al., 2006; Nefs, 2008). Surface structure affects binocular disparities in both space and shading. The VBE is one of several lines of evidence that vision uses both dimensions of information.

### IMAGE INTENSITIES AND SPATIAL POSITIONS CO-VARY

Monocular image structure involves spatial variations of intensity. Regardless of one’s representation of the physical dimensions, *space,* and *intensity* are not visually independent.

The spatial position of a given optical feature (e.g., edge) can be represented relative to an independent reference frame or topologically, relative to the surrounding image structure. Examples of both approaches are common in vision science. The concept of binocular disparity often involves the intuitive concept of space as independent of the objects and patterns it contains. Intuitively, retinal anatomy might provide such spatial coordinates.

Alternatively, the topology of spatial relations at a given point may be described in several ways. Topological parameters include (a) complexity (number of points or regions), (b) dimensionality, and (c) scale (size of neighborhood).

A familiar topological description is Fourier analysis. The Fourier power spectrum involves correlations between image contrasts at pairs of points. The Fourier phase spectrum specifies relative positions of various wavelengths, involving relations among triples of points (Yellott, 1993). The phase spectrum is essential to most aspects of visible image structure, including stereopsis (Piotrowski and Campbell, 1982; Smallman and McLeod, 1994; DeAngelis et al., 1995; Blake and Wilson, 2011). The power and phase spectra are translation-invariant. Neither requires retinal coordinates.

Another topological description is based on differential geometry. Koenderink and van Doorn (1976, 1992a,b, 1997) and Koenderink (1986, 1990) are chiefly responsible for developing the differential geometry of image structure.

The spatial structure of image intensity provides visible information about variations in surface orientation relative to both viewing and illumination directions. Countless illustrations are found in literatures on image shading in photography, painting, computer vision, and vision science (e.g., Koenderink and van Doorn, 2004). Evidently, the VBE also illustrates such effects.

The VBE shows that retinal position disparity is not necessary for stereopsis. Other experiments reviewed below show that disparate retinal positions are also insufficient.

### PERCEIVED SURFACE SLANT IS IMPRECISE

Perceived depth in the VBE seems smaller, less compelling, and less reliable than that from disparate spatial positions.

Is stereopsis simply insensitive to intensity disparities? Actually, binocular vision seems quite sensitive to dichoptic contrast differences; and these contrast differences affect perceived spatial positions in binocularly fused images (Ding and Sperling, 2006).

One source of variable perceived surface slant in the VBE is that dichoptic intensity differences have two complementary perceptual effects—on binocular brightness as well as depth rotation (Hetley and Stine, 2011). Hetley and Stine (2011) found that the relative magnitudes of these two effects varied between observers and conditions, but the combined effect was relatively constant.

Another limitation of the VBE is that surface slant is not reliably perceived anyway—from binocular disparity, structure-from-motion, image shading, texture, or other information. This perceptual limitation is hardly surprising: image information about surface orientation necessarily depends on the observer’s viewing position. Experimental evidence about the imprecision of stereoscopic slant perception is reviewed below (Section Stereoscopic Surface Slant is Imprecise).

## STEREOSCOPIC DEPTH PERCEPTION

To identify input information for stereopsis, one can work backwards from perceptual output to optical input: What structure of binocular disparity is necessary and sufficient for perceiving environmental structures in depth?

This strategy exemplifies means-end analysis (Simon, 1996) and Gibson’s (1966) method in *“The senses considered as perceptual systems.”* This method is common in engineering, but it differs from starting with presumed retinal input. A difficulty with the conventional input-first approach is that binocular disparity and optical information can be represented in many ways. Few representations suffice for stereoscopic perception, however.

Stereopsis is not necessary for perceiving a 3D world, but visual experience is much clearer with stereopsis than without it. Differences in perception with and without stereopsis are subjectively profound, as described by Oliver Sacks (“Stereo Sue,” in *The mind’s eye*, Sacks, 2010) and Bruce Bridgeman (http://www.bbc.com/future/story/20120719-awoken-from-a-2d-world).

Moreover, stereopsis greatly improves spatial acuity. Acuity thresholds for binocularly disparate relative positions are about 25% of those for the same patterns without disparity (Berry, 1948; Westheimer and McKee, 1979; Lappin and Craft, 2000).

What, then, is the structure of stereoscopic perception? Is *depth* a perceptually created third dimension? That is a common intuition, but not the only possibility.

Alternatively, stereoscopic space and depth may derive from visible relations among objects. Several hypotheses are possible about the primitive visual topology of perceived space.

Experimental research indicates that *surface shape* is an elementary visual property. From traditional perspectives, this conclusion is very counter-intuitive. Higher-order object structures would seem to derive from simpler visual cues.

Contemporary understanding of the visual role of surfaces and surface shape is due chiefly to Koenderink and van Doorn (1992a,b, 1997) and Koenderink (1990). Basic theoretical results include: (1) Environmental object surfaces and their retinal images are both 2-dimensional manifolds, described at any point by spatial derivatives in two principal orthogonal directions. (2) The differential structures of environmental surfaces and the binocular disparity fields of their images are approximately isomorphic. (3) Image information about local surface shape is given by the 2nd-order differential structure of the image fields of binocular disparity and motion parallax, which specify the ratio of minimum and maximum curvature at each position. (4) 2nd-order image information about local surface shape can be estimated directly without first estimating lower-order properties such as depth or surface orientation. (5) Variations in local surface shape are invariant with depth, slant, and curvedness.

Before examining experimental evidence, consider alternative hypotheses about perceived absolute and relative depths.

### ABSOLUTE DEPTHS OF INDIVIDUAL POINTS ARE VISUALLY UNDEFINED

The simplest spatial primitive is an individual point. Spatial positions and binocular disparities of points might be visually defined by retinal anatomy. This is a common intuitive conception.

Nevertheless, a single point is generally recognized as stereoscopically ambiguous without a reference point at fixation (Howard and Rogers, 2002).

Binocular alignment of the two retinal coordinate systems is problematic, however, because alignment varies substantially with the direction and distance of gaze — see Howard and Rogers (1995, 2002). Alignment is also perturbed by disparate eye-movements (Steinman et al., 1985; Ferman et al., 1987; Collewijn and Erkelens, 1990).

Despite these misalignments, the perceived 3D structure of the world usually appears constant under changes in gaze direction and distance. This perceptual stability conflicts with the hypothesis that stereoscopic depth derives from retinal positions. Moreover, stereo acuity thresholds for relative position are robust under disparate motions of the monocular images (Westheimer and McKee, 1978; Steinman et al., 1985; van Ee and Erkelens, 1996; Lappin and Craft, 1997, 2000). Thus, stereoscopic depth cannot derive from disparities in retinal positions of individual points.

### PERCEIVED DEPTH DIFFERENCES ARE IMPRECISE

An alternative hypothesis is that stereopsis provides perception of depth differences between pairs of points.

The retinal separation between two points and associated binocular disparity is invariant with the locus of fixation. But the relation between pair-wise image disparity and physical depth difference still depends on distance of the objects from the observer. When viewing distance, *D*, is large relative to the inter-ocular separation, *I*, then for a given disparity (in pair-wise separation), ∂, the corresponding depth difference, *Δd*, increases approximately with the square of the viewing distance:

(1) $$\Delta d\approx (D^{2}/I)\partial$$

This strong influence of viewing distance is a fundamental limitation of pair-wise disparities. As expected, perceived depth differences are unreliable.

Studies by McKee et al. (1990) and Norman et al. (2008) found that perceived depth differences between two objects were imprecise, as quantified by large Weber fractions. McKee et al. (1990) found thresholds for stereoscopic depth differences about 3–5 times higher than those for monocular separations of the same stimuli. Norman et al. (2008) found similar imprecision, with Weber fractions (coefficient of variation = *SD/M*) ∼22%. In contrast, Weber fractions for simply detecting depth are less than 0.5% (e.g., Lappin and Craft, 1997, 2000).

### STEREOSCOPIC SURFACE SLANT IS IMPRECISE

Koenderink and van Doorn (1976) and Koenderink (1986) showed that surface slant affects the “deformation” component of the 1st-order spatial derivatives of the binocular disparity field — involving disparate shapes of triangular surface patches. The deformation component is invariant with image translation, expansion, and rotation, but it varies with viewing direction and distance (see Howard and Rogers, 2002, chap. 21). Accordingly, perceived surface slant is ambiguous.

Slant detection is also anisotropic, because the eyes are horizontally separated, with more sensitivity to vertical than horizontal disparity gradients (Rogers and Graham, 1983; Gillam and Ryan, 1992).

The predictable unreliability of slant discriminations has been found experimentally (e.g., Todd et al., 1995). Current evidence is limited, however: judgmental reliability is often not reported; viewing distance and context are often constant; and disparity gradients usually co-vary with texture gradients and other information.

Experiments by Norman et al. (2006, 2009) found that stereopsis adds very little to the limited precision of slant estimates based on texture, relative motion, and shading. Surfaces in both studies were seen at a constant distance; and judgments would have been less precise with varied viewing distances.

Steep surface slants may be difficult to discriminate or even detect when disparity changes too much in too small an area. Filippini and Banks (2009) evaluated stereoscopic detection of large depth gradients, using random-dot saw-tooth surfaces in noise. Signal/noise thresholds for surface detection rose rapidly for disparity/separation ratios above 1.0, as predicted by cross-correlation models.

Other experiments, however, have found that depth changes on smooth surfaces are more visible than predicted by a cross-correlation model. Allenmark and Read (2010) found that large depth changes were as visible on smooth sine-wave surfaces as on square-waves. Norman et al. (1991) found very accurate discriminations of surface *smoothness*, exceeding predictions of cross-correlation or other linear models.

### SURFACE SHAPE IS A PERCEPTUAL PRIMITIVE

Human observers can discriminate very small variations in surface shape — with greater precision than for discriminations of depth or slant, and invariant under random perturbations of depth and slant(e.g., van Damme and van de Grind, 1993; Todd et al., 1996, 1997; Perotti et al., 1998; Lappin and Craft, 2000; Todd, 2004; Lappin et al., 2011).

Norman et al. (1991) found accurate perception of surface smoothness. Random-dot triangle-wave surfaces, discontinuous at their extrema, were discriminated from very similar smooth surfaces (fundamental + 3rd harmonic of the triangle-wave) with slight curvature at the extrema. Smoothness discriminations were more accurate than detections of the differences in Fourier power spectra. Thus, stereoscopic perception yielded curved surfaces (2nd-order structure), not depths or slants.

Shape discriminations are more reliable than and independent of perceived depth differences (van Damme and van de Grind, 1993; Todd et al., 1996, 1997; Perotti et al., 1998; Todd, 2004). Smooth surface shape, therefore, is a fundamental visual property not derived from perceived depths or slants.

## BINOCULAR DISPARITY

What does stereoscopic perception tell us about binocular disparity, the input information for stereopsis?

### DISPARITY INVOLVES IMAGE STRUCTURE

The first principle is that stereoscopic input involves disparate image structures, not disparate retinal positions. Stereoscopic hyper acuity (resolution finer than the eye’s photoreceptor density, point spread function, and diffraction limit) is robust under random perturbations of retinal image positions in each eye (Sections Absolute Depths of Individual Points are Visually Undefined and Surface Shape is a Perceptual Primitive). Thus, monocular spatial positions are visually defined relative to the surrounding image.

### DISPARITY INVOLVES SURFACE SHAPE

Stereoscopic vision is directly sensitive to the shapes of environmental surfaces (Section Surface Shape is a Perceptual Primitive). Surface shape is discriminated more reliably than seemingly simpler properties; and hyper acuity for surface shape is maintained under random perturbations of lower-order disparities associated with relative depth and slant (Norman et al., 1991; Perotti et al., 1998; Lappin and Craft, 2000).

Stereoscopic perception of surface shape is possible because of structural correspondences between environmental surfaces and binocular disparities—involving 2nd-order spatial derivatives (Koenderink and van Doorn, 1992a; Lappin and Craft, 2000; Todd, 2004; Lappin et al., 2011).

### DISPARITY OF 2ND-ORDER IMAGE STRUCTURE

The “2nd-order differential structure” of binocular disparity is simpler than it might first seem. The relevant structure is just the radial symmetry of the neighborhood around every local image point. The disparate binocular images of a surface differ by a deformation of this symmetry. The qualitative form of this local image deformation corresponds to the local surface shape, invariant with the observer’s viewing position.

**FIGURE 1:**
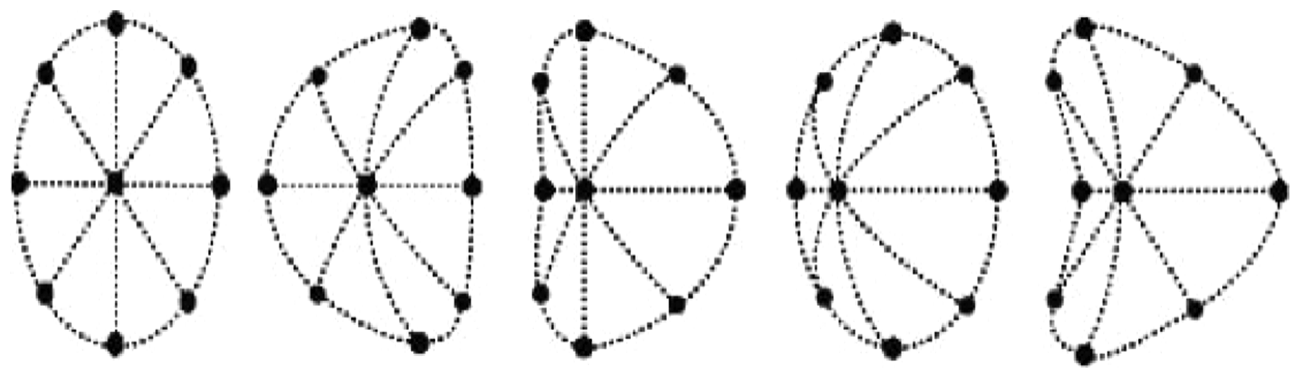
**Schematic forms of image deformations produced by rotating the viewpoint of a circular surface patch around its central vertical axis.** Rotation direction and concavity vs. convexity are ambiguous. The shapes, from the left, are *planar* (0 curvature), *parabolic* (0 curvature in one axis), *parabolic*, *elliptic* (with the same sign of curvature in both axes), and *hyperbolic* (opposite signs of curvature in the two axes; Illustration from Lappin and Craft, 2000, Figure 3, p. 14. Copyright 2000 by the American Psychological Association. Reprinted with permission).

**Figure 1** illustrates these image deformations for each of the possible surface shapes. As may be seen, these stereo deformations correspond, from left to right, to local images of a plane, horizontal cylinder, vertical cylinder, ellipsoid, and saddle—as specified by the relative magnitudes of the two principal curvatures (horizontal and vertical in this illustration). These patterns exemplify the qualitative possibilities for smooth surfaces.

**FIGURE 2:**
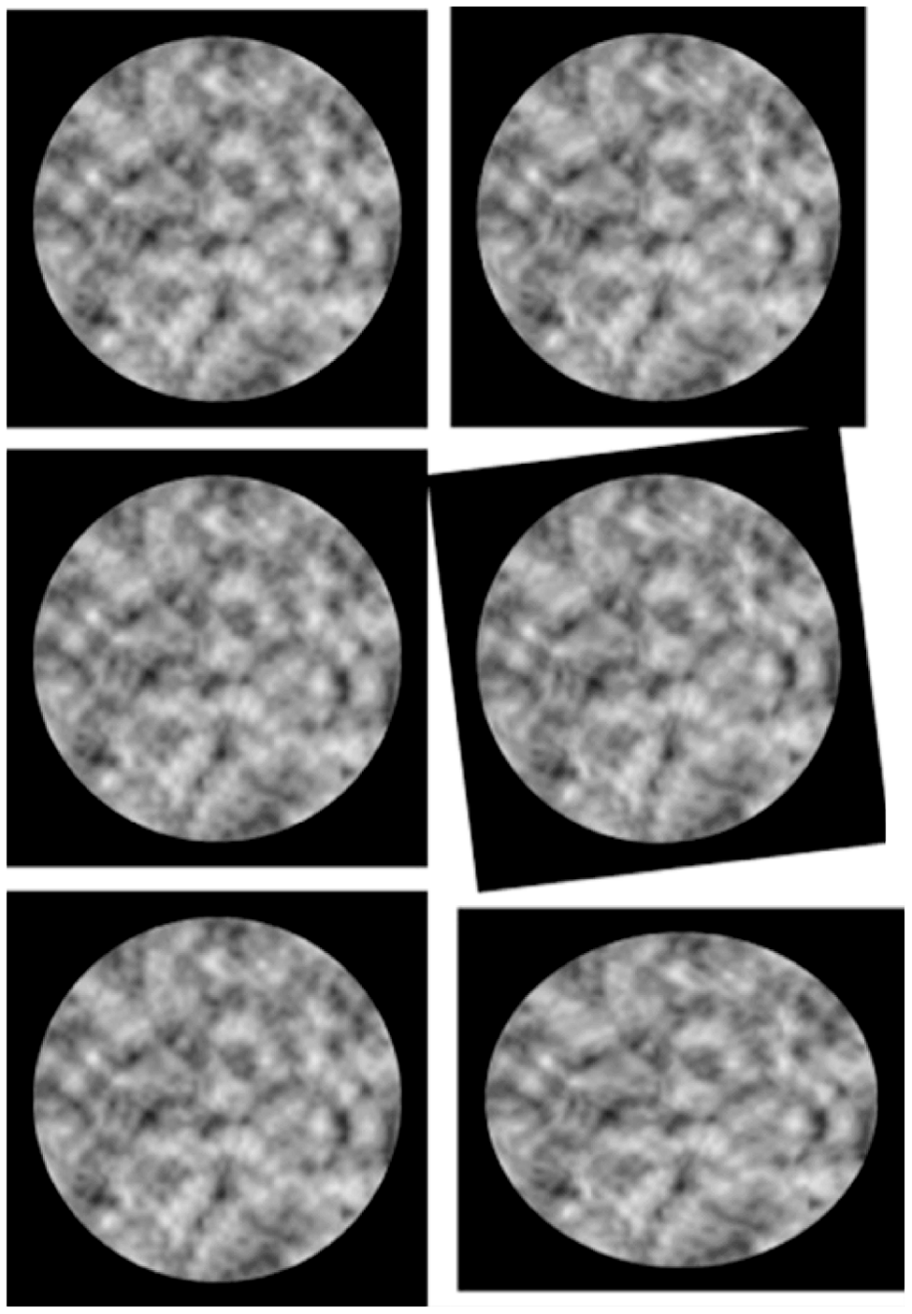
**Stereo illustrations of perceived shape from binocular disparity, invariant under global image transformations by 2D rotation and shear.** Shape and shading are random and mutually independent. Top: undistorted stereo, with right image rotated in depth around the vertical axis by about 5°. Center: right image rotated about 7°. Bottom: right image expanded and compressed by about 7% in orthogonal axes (“pure shear”). The left image is identical in all three pairs. (Illustration from Lappin et al., 2011, Figure 10, p. 2368. Copyright 2011 by the Psychonomic Society. Reuse of this illustration with kind permission from Springer Science+Business Media.)

**Figure 2** demonstrates the robust visual sensitivity to smooth variations in these local structural disparities in images of randomly shaped surfaces. Image information about local surface shape is preserved under significant global disparity changes produced by rotating, dilating, or shearing the image plane—as illustrated by the middle and lower panels. Like most random-element stereograms, the random intensities in these patterns are independent of surface shape and binocular disparity; but here depths and intensities both vary smoothly, without sharp edges. Unlike most natural images, shading is unrelated to surface shape; and the intensities are not disparate.

### BINOCULAR DISPARITY IN THE VENETIAN BLIND EFFECT

The VBE involves perceived rotation of vertical bars. Such planar rotations ordinarily produce bilaterally symmetric dilation or compression of horizontal scale, as seen at the left of **Figure 1**. Changing the horizontal distribution of relative intensities may have similar effects on visual neurons responsive to the left-right balance of surrounding stimulation. Perceived rotation seems a plausible and understandable result of this image disparity.

As Dobias and Stine (2012) note, the explanation for the direction of perceived rotation is not immediately obvious. Image shading from reflective surfaces depends on illumination direction as well as surface orientation. For special cases, however, with Lambertian shading (equal scattering in all directions), radiant surfaces, and surfaces illuminated from behind, image intensity is greater when the surface is perpendicular to the viewing direction. Thus, the surface orientation may plausibly appear more perpendicular (and thus expanded) toward the eye with greater relative intensity or contrast.

In general, stereoscopically perceived surfaces derive from binocular disparities of higher-order image structures. For the visual system, spatial position and intensity are correlated dimensions. Relative spatial positions involve relative intensities. Both are structured by surfaces, and both constitute information about surface structure, not depth as such.

## Conflict of Interest Statement

The author declares that the research was conducted in the absence of any commercial or financial relationships that could be construed as a potential conflict of interest.
